# Comparative study of alginate and type I collagen as biomaterials for cartilage stem/progenitor cells to construct tissue-engineered cartilage *in vivo*

**DOI:** 10.3389/fbioe.2022.1057199

**Published:** 2023-01-11

**Authors:** Xiaodie Zhang, Lin Qi, XiaoGang Chen, Yongxian Lai, Kai Liu, Ke Xue

**Affiliations:** ^1^ Department of Dermatologic Surgery, Shanghai Skin Disease Hospital, Tongji University School of Medicine, Shanghai, China; ^2^ Department of Radiology, Huadong Hospital Affiliated to Fudan University, Shanghai, China; ^3^ Department of Plastic and Reconstructive Surgery, Shanghai 9th People’s Hospital, Shanghai Jiao Tong University School of Medicine, Shanghai, China; ^4^ Department of Burn and Plastic Surgery, Hainan Western Central Hospital, Shanghai, China

**Keywords:** alginate, type Ⅰ collagen, cartilage, cartilage stem/progenitor cells, tissue engineering

## Abstract

With the help of biomaterials, cartilage stem/progenitor cells (CSPCs) derived from cartilage tissue present a promising choice for cartilage regeneration. In our previous study, we investigated whether CSPCs could be ideal seeding cells for cartilage tissue regeneration. Biomaterials are fabricated to accelerate tissue regeneration, providing a suitable environment for cell attachment, proliferation, and differentiation. Among the biomaterials used in cartilage regeneration medicine, alginate and collagen are classified as natural biomaterials and are characterized by high biocompatibility, bioactivity, and non-toxic degradation products. However, it is unclear which material would have a competitive advantage in CSPC-based cartilage regeneration *in vivo*. In the present study, we employed alginate and type Ⅰ collagen as substrates for CSPCs and chondrocytes, which was made control group, to explore a more suitable biomaterials for CSPCs to fabricate tissue-engineered cartilage, *in vivo*. Hematoxylin and eosin (HE) staining, Safranin O, immunohistochemical assay, and quantitative real-time polymerase chain reaction (qRT-PCR) were used to evaluate the tissue-engineered cartilage *in vivo*. Compared with the alginate group, collagen enhanced the expression of cartilage-specific genes, such as ACAN, SOX9, and COLII, more markedly. Furthermore, the marker genes of expression, dedifferentiation, and hypertrophy, COLI and COLX, were downregulated in the collagen group. The results demonstrated that collagen as a substrate was superior to alginate in increasing the accumulation of cartilage-like ECM for CSPCs *in vivo*. In summary, compared with alginate, collagen hydrogel is an effective biomaterial for CSPC-based cartilage regeneration.

## Introduction

Due to its poor regenerative capacity, cartilage rarely produces effective regeneration, which causes patients pain and limitation in their usual activities of daily life (Kilmer et al., 2020; Maihofer et al., 2021; Cui et al., 2023). Cell-assisted therapy has demonstrated promising results for cartilage regeneration (Li et al., 2020; Xue et al., 2021; Gu et al., 2022). Stem cells have inspired research due to their special biological structure and characteristics at the molecular level (Li et al., 2020; Rodriguez Ruiz et al., 2021; Palmquist-Gomes et al., 2022). The most prominent features of stem cells are their strong capacity for self-renewal and their biological plasticity. Researchers have successfully isolated different types of stem cells from various tissues, and most scholars believe that there should be a certain proportion of cartilage stem cells in normal cartilage tissue to maintain a stable phenotype and biological homeostasis of the cartilage tissue (Li et al., 2020; Rodriguez Ruiz et al., 2021; Gu et al., 2022). Interestingly, defective auricular cartilage tissue has been shown to reproduce in patients who received ear cartilage augmentation rhinoplasty without coming into contact with the auricle perichondrium. Similar results were observed when rat perichondrium was transplanted into articular cartilage defects (Dou et al., 2021). These observations suggest that there might be a group of stem cells in cartilage tissue or the surrounding tissue that could be responsible for complete regeneration of cartilage tissue through proliferation and differentiation. In 2004, Alsalameh et al. demonstrated that human cartilage tissue contained subgroups of cells with biological characteristics and behaviors similar to mesenchymal stem cells (Alsalameh et al., 2004). In the following decades, more research about cartilage-derived stem cells was reported (Kobayashi et al., 2011; Embree et al., 2016; Zhang et al., 2019). Our team successfully isolated a subpopulation of stem/progenitor cells from the ear cartilage of newborn piglets, named cartilage stem/progenitor cells (CSPCs) (Xue et al., 2015; Xue et al., 2019). When compared with bone-derived stem cells, CSPCs exhibited greater chondrogenic potential but lower osteogenic and angiogenetic capacity, which indicated they would be a suitable alternative cell source for cartilage tissue engineering or cartilage regeneration medicine.

Biomaterials play an important role in cartilage regeneration (Pustlauk et al., 2016; Wei et al., 2021). The ideal scaffold material should have the following characteristics: good biodegradability, as the degradation rate of the scaffold material should be compatible with the cell growth rate; good biological compatibility, which is conducive to seed cell adhesion, proliferation, growth, and differentiation; and a porosity that facilitates the migration of cells and is evenly distributed on the scaffold. Furthermore, the scaffold should be conducive to the diffusion of nutrients, the discharge of metabolic waste, and should provide a stable external environment for cell growth and differentiation. In addition, the scaffold material should have good shapeability and it should be easy to prepare the tissue model of irregular defects (Carvalho et al., 2021; Wei et al., 2021). Currently, sodium alginate and collagen are two of the most widely used hydrogels in cartilage tissue engineering. When exposed to divalent cations, such as Ca^2+^, Ba^2+^ or Sr^2+^, alginate generates a hydrogel that is similar to an extracellular matrix (Ahmad Raus et al., 2021; Carvalho et al., 2021). Levato et al. (2017) found that employing hydrogel as a bio-scaffold material could accelerate chondrogenesis and inhibit osteogenesis in CSPCs. Moreover, Zhikang et al. indicated that alginate could accelerate chondrocyte proliferation and enhance the accumulation of cartilage matrix (Miao et al., 2018). Its chondrogenic properties and chemical characteristics make alginate an ideal alternative biomaterial to be employed in cartilage tissue engineering and cartilage regeneration medicine.

Collagen, another type of biological scaffold material, has many advantages, such as low immunogenicity, excellent biodegradability, and excellent biocompatibility (RezvaniGhomi et al., 2021). The main type of collagen is type I collagen, which is widely found in tissues such as dermal tissue, bone, and tendon (Bielajew et al., 2020). It is formed by three strands of polypeptide chains intertwined and entangled to form collagen fibers. The current methods for separating, extracting, and purifying collagen at home and abroad include neutral salt extraction, acid separation, alkali extraction, and enzymatic digestion (Adamiak and Sionkowska, 2020). As a biological scaffold material, type I collagen not only facilitates cell adhesion, but it also promotes cell migration (RezvaniGhomi et al., 2021). In addition, type I collagen resources are extensive, its structure is in equilibrium, and its preparation is simple. Moreover, its biological advantages have been confirmed in corresponding clinical applications. It is currently a U.S. Food and Drug Administration (FDA)-certified biosafety material and has become one of the ideal bionic materials in cartilage tissue engineering. However, the effectiveness of hydrogels on CSPCs has seldom been reported, and comparison of the hydrogels *in vivo* has not been studied.

In the present study, we employed alginate and collagen as cell substrates to study their effects on CSPCs chondrogenesis and evaluated CSPCs ectopic formation *in vivo*.

## Materials and methods

### Isolation and culture of CSPCs

This experiment was approved by the Ethics Committee of the ninth Peoples’ Affiliated Hospital, School of Medicine, Shanghai Jiaotong University. Primary CSPCs from neonatal pigs (Chang Feng hybrid pig purchased from Shanghai Chuansha Breeding Factory) were obtained and characterized as in a previous study. Following previously described methods, CSPCs and chondrocytes were obtained through differential adhesion to fibronectins. (Xue et al., 2019).For the harvest of primary CSPCs, cartilage from newborn pig ears was sectioned with a scalpel in a sterile environment into 1 mm^2^ slices. Then, samples were washed with normal saline three times, digested at 37°C for 8 h in 0.2% (w/v) collagenase NB4 (Serva, Heidelberg, Germany), and dissolved in high-glucose Dulbecco’s modified Eagle’s medium (DMEM). The suspension was then filtered across a 200-µm filter to remove undigested samples and centrifuged at 1,500 rpm for 5 min. The cell pellet was resuspended and plated onto a fibrin-coated culture dish to a density of 1–2 × 10^5^ cells/cm^2^. After incubation for 20 min, non-adherent cells and media were discarded, and low-glucose DMEM containing 10% fetal bovine serum (FBS) was added to the plates. Sub-culturing followed, and the cells were cultured for approximately 2 weeks until they reached 80–90% confluence. In our previous experiment, flow cytometric analysis revealed that the cell populations expressed mesenchymal stem cell-positive surface markers, and the cells occupied multi-directional differential capacity under different induction. For the harvest of chondrocytes, the non-adherent cells and media was exposed to centrifugation at 1,500 rpm for 5 min. The cells were seeded at 5*10^3^ cells/cm^2^, and expanded in complete H-DMEM (Gibco-BRL).

### Cell and biomaterial construction

For CSPCs undergoing type I rat tail collagen formation, the third passage of the CSPCs was collected and the cell concentration was adjusted to 50 × 10^6^ cells/ml. The cells were then placed on ice until use. We added 12 µl of prepared 100 mM NaOH solution to 200 µl of type I rat tail collagen (Solario, C8062) and mixed rapidly. Then, 23 μl of precooled, sterilized 10× PBS was added into the mixture, and 760 μl of the cell suspension was combined with the mixture. The solution on ice was mixed lightly and rapidly to avoid the generation of bubbles, and 200 μl of the solution was transferred to a new sterile 15 ml centrifuge tube. Following the addition of 3 ml of preheated low-glucose DMEM, the tube was kept in a constant-temperature incubator for 15 min to gel. The constructed CSPCs/type I rat tail collagen were incubation at 37°C in an atmosphere of 5% CO_2_. Then, the culture medium was gently replaced with new complete L-DMEM.

For the CSPCs/alginate group, a 2% (w/v) sodium alginate solution was pretreated by dissolving alginic acid sodium salt (low viscosity; Sigma) in deionized water, and the solution was strained through a 0.22 um filter (Millipore). Before being extruded into the 0.1 M CaCl_2_ solution with a 23 G needle, the sterile alginic acid sodium salt solution was mixed gently with the CSPCs suspension (50 × 10^6^ cells/ml) and crosslinked for 10 min to obtain CSPCs/alginate beads. The CSPCs/alginate beads were washed with preheated L-DMEM without FBS and incubated in L-DMEM containing 10% FBS at 37°C and 5% CO_2_. The CSPCs/alginate beads and CSPCs/type I rat tail collagen were incubated with chondrogenic induction medium (10% FBS, 10 ng/ml transforming growth factor-1, 10^−7^ M dexamethasone, and 40 ng/ml insulin-like growth factor-1 were contained in H-DMEM) for 3 weeks before transplantation into the subcutaneous tissue of an athymic mouse, and the culture medium was changed every other day. At the same time, the control groups were cultured with complete L-DMEM for 3 weeks, and the medium was changed every other day. The samples were obtained at 4 weeks and were pretreated for histological and immunofluorescence examination.

### Histological and immunofluorescence analysis

The deposition of cartilage-specific matrix in the CSPCs/biomaterial construction was assessed on paraffin-embedded samples. Tissue sections (5 µm) were sliced with a microtome and processed for staining. Hematoxylin and eosin (HE) and Safranin O staining were employed to evaluate the histological structure and visualize cartilage-specific matrix accumulation. The tissue sections (2–4 µm) were prepared and processed for immunofluorescence staining as described in the methods (Im et al., 2019). The deposition of collagen types I, II, and X was evaluated using the appropriate primary anti-collagen type I antibody (1:1,500; ab90395), anti-collagen type II antibody (1:200; ab34712), and anti-collagen type X antibody (1:2000; ab49945), respectively. Following addition of 0.3% v/v H_2_O_2_ to block endogenous peroxidases, sections were deparaffinized with xylene and hydrated in ethanol graded solution in turn. After incubation with a primary antibody overnight at 4°C, samples were exposed to anti-rat and anti-rabbit secondary antibodies (1:4,000 in 0.5% BSA; Wuhan Guge Biological Technology Co., Ltd.) for 1 h at room temperature in the presence of a nuclear staining reagent (HY-15558; MCE). Finally, images were recorded using a fluorescence microscope (Leica DMR, Germany).

### Gene expression

Four weeks after *in vivo* transplantation, samples were harvested and total RNA was extracted from every specimen. Followed previously described methods, cDNA was obtained by reverse transcription and gene expression was evaluated by real-time quantitative PCR analysis with the brilliant SYBR green qPCR kit (Strata gene, United States) (Xue et al., 2019; Xue et al., 2021). SOX9, COL2, and aggrecan were employed to evaluate chondrogenic differentiation. The primers used in our study are shown in Table 1. The level of β-actin mRNA was analyzed as an internal control. Every experiment was repeated at least three times. Glyceraldehyde 3-phosphate dehydrogenase (GAPDH) was chosen as an endogenous control to calculate relative gene expression levels.

**TABLE 1:** Primer sequences for RT-PCR.

Gene	Primer
COL1A2	Forward primer	5’- GGT​TTC​GGC​AAA​GTT​GGA​GG -3’
	Reverse primer	5’- GCC​CTT​TCT​TGC​AGT​TGC​C -3’
COL2A1	Forward primer	5’- CGA​GAC​AGG​TGC​TGC​AAG​TC -3’
	Reverse primer	5’- TGA​TCA​CCT​GGT​TTC​CCA​CC -3’
SOX9	Forward primer	5’- CTC​AGC​AAG​ACT​CTG​GGC​AA -3’
	Reverse primer	5’- TTG​GGA​GAG​ATG​TGC​GTC​TG -3’
RUNX2	Forward primer	5’- CCA​GCA​GCA​CTC​CAT​ACC​TC -3’
	Reverse primer	5’- ACG​CCA​TCG​TTC​TGG​TTA​GG -3’
ACAN	Forward primer	5’- CTC​ACG​GTG​AAA​CCC​GTC​TT -3’
	Reverse primer	5’- TCGGGAAAAGCCCAGGGT -3’
MMP13	Forward primer	5’- ATGAATCCTGCTGGAATC -3’
	Reverse primer	5’- CATTTGGGACCATTTGAG -3’
COL10A1	Forward primer	5’-TGC​TGC​TAT​TGT​CCT​TGA​AC -3’
	Reverse primer	5’- ATA​CCT​TGC​TCT​CCT​CTT​AGT​G -3’
GAPDH	Forward primer	5’- CCT​CAA​CGA​CCA​CTT​CGT​CA -3’
	Reverse primer	5’- GGG​TCT​GGG​ATG​GAA​ACT​GG -3’

### Statistical analysis

Data are expressed as mean ± standard deviation, and statistical analysis was performed using SPSS software version 15.0 (IBM Corp, Armonk, NY). Every group had a sample size of at least three replicates (n = 3). Statistical significance was determined by performing a one-way ANOVA followed by a Tukey’s post-hoc test. *p* < 0.05 was considered statistically significant.

## Results

### Gross assay of hybrids formed by CSPCs/biomaterials

**FIGURE 1:**
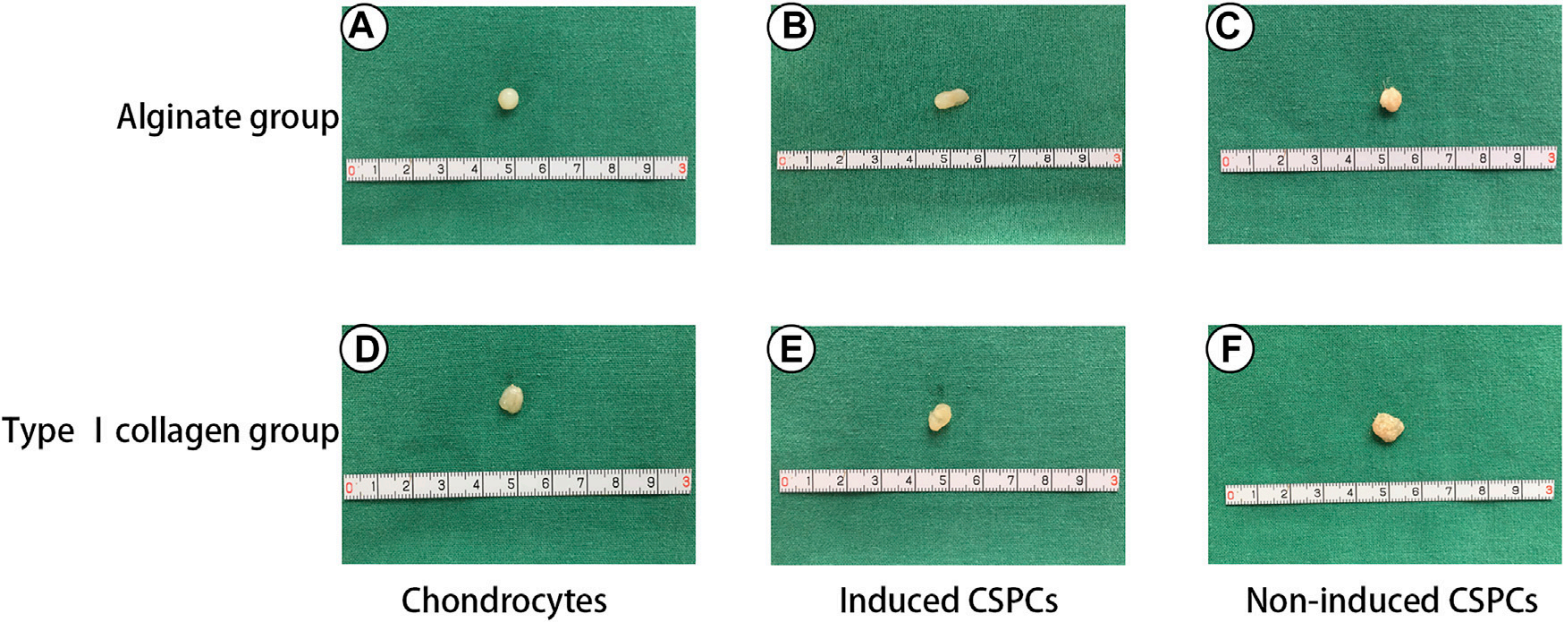
Gross assay of constructs formed by chondrocytes/alginate and CSPCs/type Ⅰ collagen. All the chondrocyte groups **(A)** and **(D)** and induced groups **(B)** and **(E)** had an ivy white appearance, which appeared similar to native cartilage tissue. In contrast, the non-induced group **(C)** and **(F)** exhibited shrinkage.

To estimate the chondrogenic morphology of the hydrogel constructs, gross assay was performed (Figure 1). Between the induced groups, both CSPCs/type I rat tail collagen and CSPCs/alginate displayed an ivory-white cartilage-like appearance (Figures 1B,E), and there were no obvious differences between them and the chondrocyte group. In contrast, between the non-induced groups (Figures 1C,F), constructs formed by CSPCs/type I rat tail collagen had a closer appearance to constructs formed by chondrocytes.

### Histological examination of CSPCs/biomaterials

**FIGURE 2:**
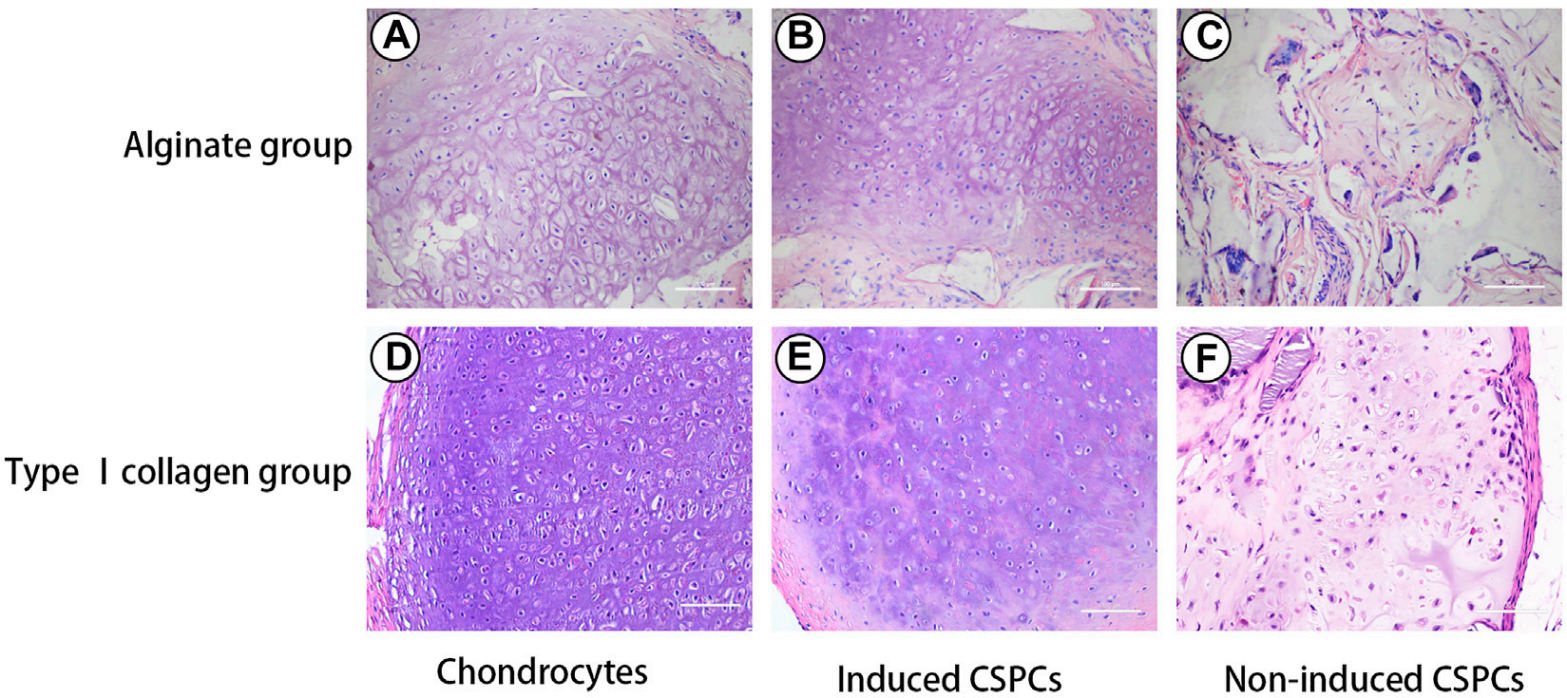
Hematoxylin and eosin (HE) staining. Some of residual biomaterials were observed in all groups **(A–F)**. Chondroid matrix in the non-induced treatment group **(C)** and **(F)** was lower in quality compared with the other groups **(A,B,D,E)**. The induced groups **(B)** and **(E)** formed mature cartilage tissue, which were closed to chondrocytes group **(A)** and **(D)**. Even though necrosis was observed in the induced and non-induced groups **(B,C,E,F)**, the induced CSPCs/type Ⅰ collagen group **(B)** displayed a more uniform cartilage-like matrix than the induced CSPCs/alginate group **(E)**. Scale bar: 100 μm.

Four weeks after the constructions were transplanted into the subcutaneous area of athymic mice, HE staining was used to evaluate chondrogenesis (Figure 2). Histological evaluation revealed that some of the residual biomaterials could been recognized in every group, and the formation of a chondroid matrix from the non-induced treatment group was of lower quality compared with the hybrids of chondrocytes/biomaterials. Although the generated cartilage tissue was uneven, the degree of accumulation of cartilage matrix in the induced groups was between that of the control group and the non-induced group, and mature cartilage tissue was observed in most areas in the two induced groups. The samples exhibited round or cobblestone-like morphology of chondrocytes, and the typical lacunar structure of cartilage was present. Furthermore, the number of chondroid cells in the induced CSPCs/type I rat tail collagen group and CSPCs/alginate group was higher than in the non-induced group. Interestingly, some cartilage-like matrix was recognized in the uninduced hybrids made of type I rat tail collagen (Figure 2F), while such a phenomenon was not observed in the non-induced alginate group (Figure 2C). Except for the chondrocyte group, necrosis occurred in the four CSPCs groups, which markedly increased inflammatory cell infiltrate, and the necrosis in the non-induced CSPCs/alginate group was more obvious than in the other CSPCs groups.

**FIGURE 3:**
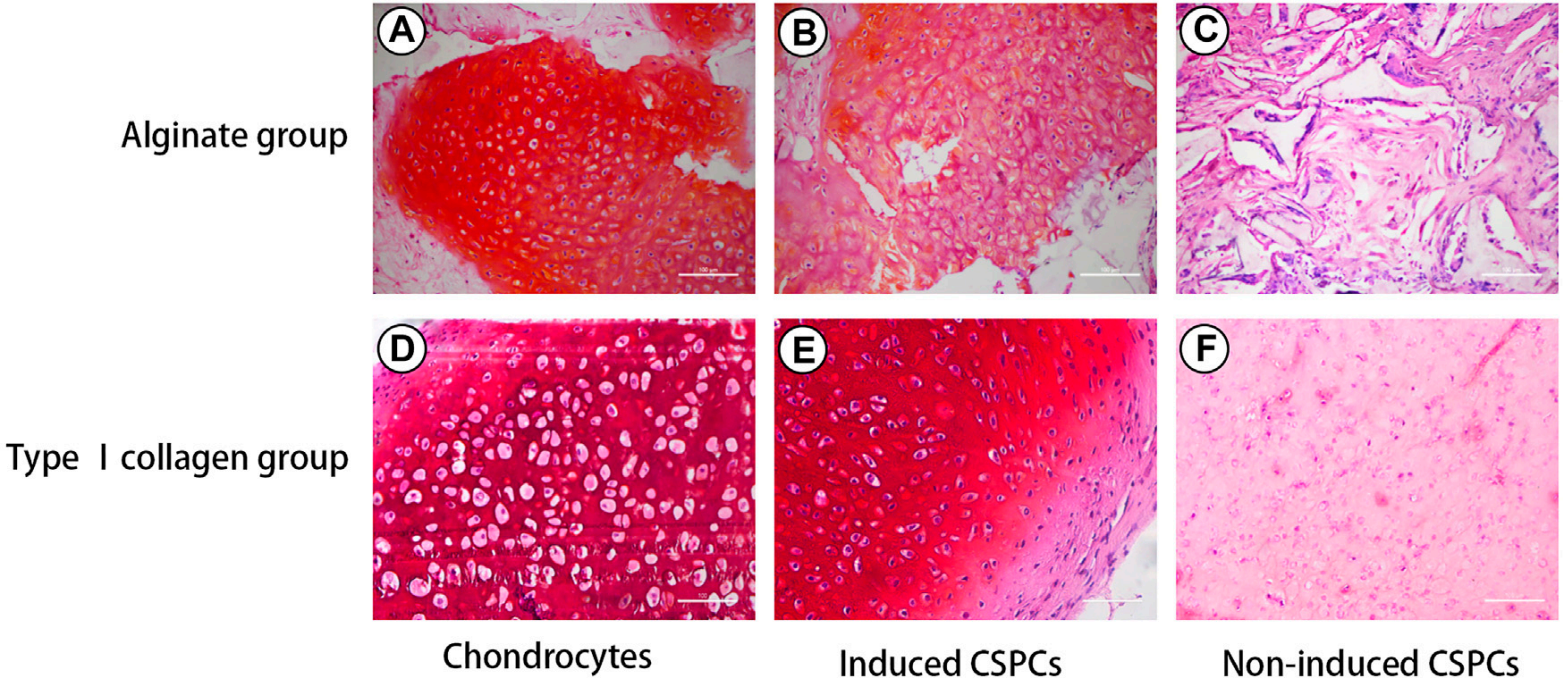
Safranin O staining. Positive Safranin O staining indicated that induced groups **(B)** and **(E)** formed cartilage-like tissue, which was also observed in the chondrocytes group **(A)** and **(D)**. The induced CSPCs/type Ⅰ collagen group (B) formed more mature cartilage-like matrix than the induced CSPCs/alginate group **(E)**. A weak positive reaction was observed in the non-induced type Ⅰ collagen group **(F)**, while positive reaction could be recognized in the non-induced alginate group **(C)**. Scale bar: 100 μm.

The results of Safranin O staining (Figure 3) were consistent with those of the HE staining and further confirmed the formation of cartilage matrix. Both the chondrocyte group and the induced group had strong signals for GAG, and the cells were surrounded by a large amount of red extracellular matrix, confirming that they differentiated more effectively into cartilage-like tissue; in contrast, the non-induced type I rat tail collagen group exhibited a weak positive reaction.

### Immunofluorescence staining of CSPCs and biomaterials

**FIGURE 4:**
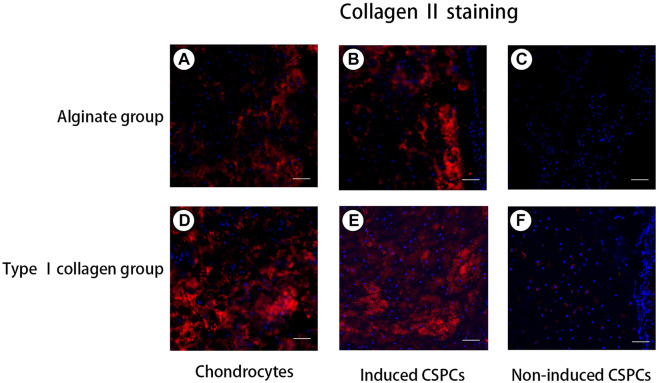
Analysis of collagen Ⅱ formation. **(A,B,D,E)**. Positive reaction was exhibited by the chondrocytes group **(A)** and **(D)** and induced group **(B)** and **(E)**, and the reaction of the induced CSPCs/type Ⅰ collagen group **(B)** was between that of the two group but was stronger than the induced CSPCs/alginate group **(E)**. A weak positive reaction was observed in the non-induced type Ⅰ collagen group **(F)**, especially around cells. However, negative reaction was presented in the non-induced alginate group. Blue: cell nucleus, red: collagenⅡ. Scale bar: 50 μm.

**FIGURE 5:**
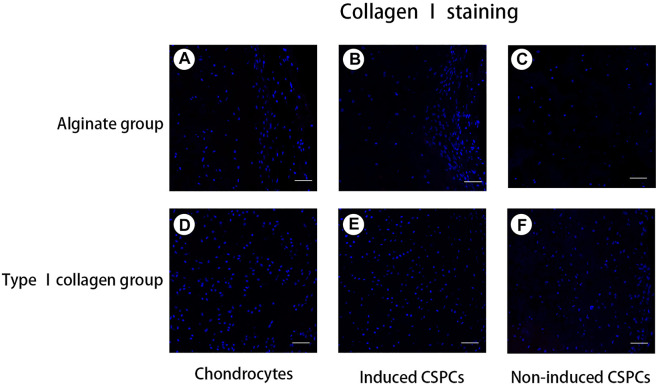
Analysis of collagen Ⅰ formation. **(A–F)**. A negative reaction was observed in every group, which indicated that the hydrogel opposed cartilage regeneration. Blue: cell nucleus, red: collagen Ⅰ. Collagen. Scale bar: 50 μm.

**FIGURE 6:**
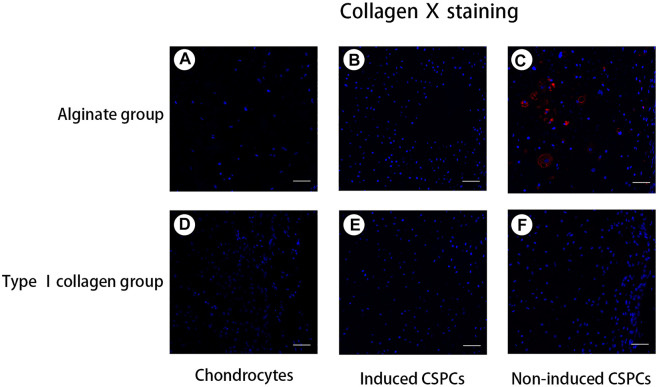
Analysis of type X collagen formation. **(A,B)** and **(D–F)**. A negative reaction was observed in the chondrocyte groups **(A)** and **(D)**, induced groups **(B)** and **(E)**, and non-induced CSPCs/type Ⅰ collagen group **(F)**. A weak positive reaction was observed in the non-induced CSPCs/alginate group **(C)**, which indicated that type Ⅰ collagen might have an inhibitory effect on hypertrophy andchondrogenic differentiation from CSPCs. Blue: cell nucleus, red: collagen Ⅹ. Scale bar: 50 μm.

To confirm the formation of cartilage-specific matrix, we used immunofluorescence detection to characterize the secretion of cartilage-specific matrix, collagen Ⅱ (Figure 4), and related matrix proteins. After 4 weeks of culture *in vivo*, the immunofluorescence of type II collagen in the chondrocytes group (Figures 4A,D) and induced group showed a positive reaction; the chondrocyte group and induced type I (Figures 4B,E) rat tail collagen group exhibited a strong positive reaction, and the degree of the positive reaction exhibited by the induced alginate group was between the other group. Collagen Ⅰ (Figure 5), a marker for de-differentiation and fibration, and collagen Ⅹ (Figure 6), signs of engineered cartilage hypertrophy, were also been exposed to immunofluorescence staining (Zhang et al., 2015). Collagen Ⅰ was not present in every group according our immunofluorescence analysis. However, immunofluorescence detection of type I collagen and type X collagen showed no obvious positive reaction in the chondrocyte group and induction type I rat tail collagen group; in contrast, type X collagen was weakly positive in the alginate non-induced group.

### qRT-PCR

**FIGURE 7:**
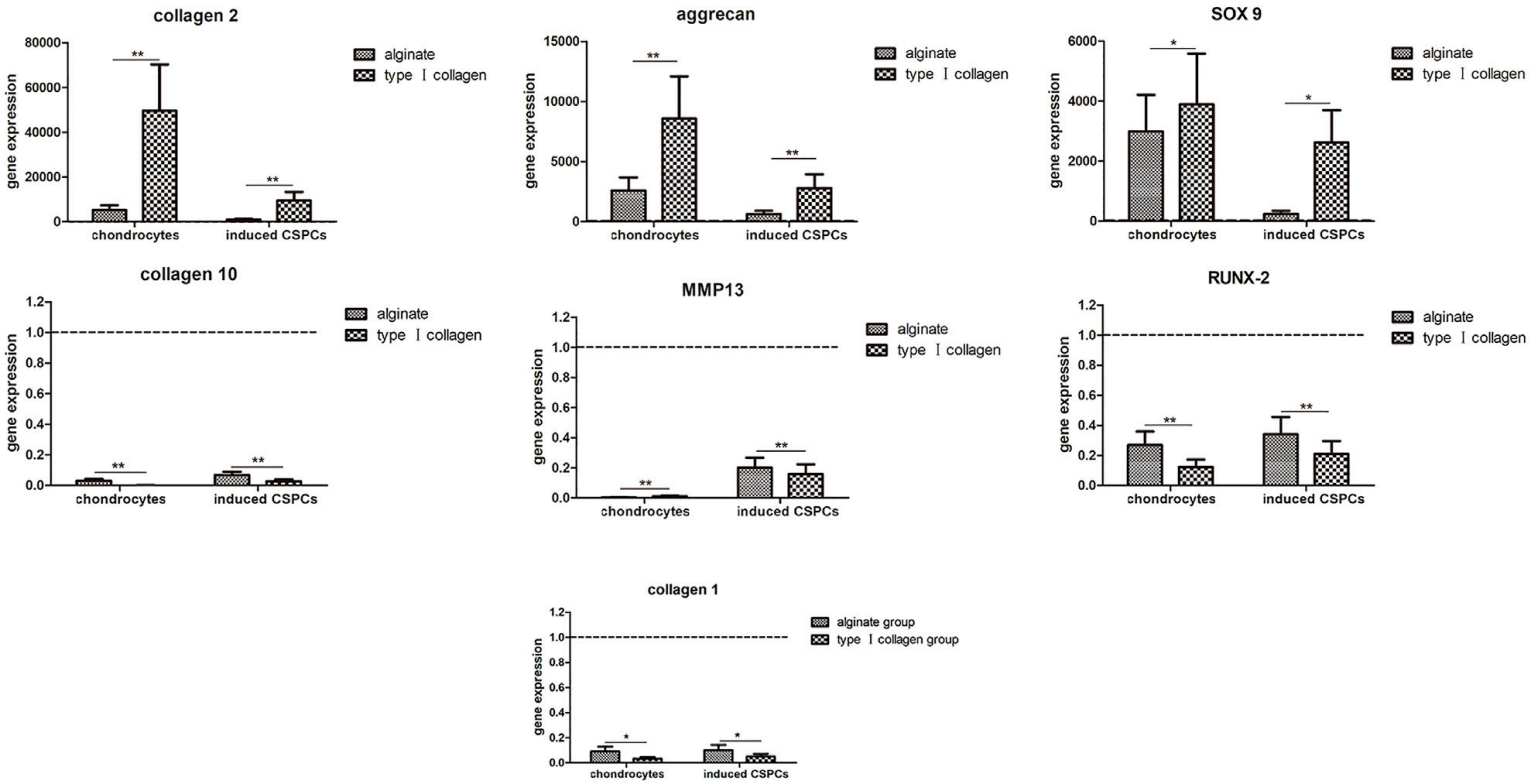
Gene expression analysis. Upregulation of type Ⅱ collagen and aggrecan and downregulation of type Ⅰ and type Ⅹ collagen were observed in the induced groups and chondrocytes groups. Similar results were found in CSPCs/type Ⅰ collagen group compared with the CSPCs/alginate group. The fold change (2-∆∆Ct) was calculated using the relative expression of non-induced CSPCs/biomaterials as a control group (indicated by the dotted line). Values are presented as mean ± standard deviation and were normalized to GAPDH. *p<0.05, **p<0.01.

We employed three cartilage-specific genes, COL2A1, SOX9, and ACAN, to visualize chondrogenesis from CSPCs/biomaterial (Figure 7). QT-PCR revealed that the expression of SOX9 and ACAN in the inducted group was close to that of the chondrocyte group, and there was a significant difference between the expression in the type I rat tail collagen and alginate group. The similar expression of COL2A1 was also observed in the three groups. The results further confirmed the histological and immunohistochemical analyses and together indicated that type I rat tail collagen could be a more effective biomaterial than alginate for tissue-engineered cartilage generated from CSPCs. Furthermore, we analyzed the expression of cartilage hypertrophy genes COL10A1, MMP13, and osteogenesis-related gene COL1A1. The results showed that the expression of COL10A1 and MMP13 in the induced group was inhibited compared with the non-induced alginate group, and there was no significant difference in the chondrocyte group. Moreover, there was no significant difference in the expression of COL1A1 between all groups. These results suggest that type I rat tail collagen had more advantages over alginate in constructing engineering cartilage with a stable phenotype.

## Discussion

Composed of a large amount of extracellular matrix and few chondrocytes with limited proliferation ability, cartilage tissue is known for its scarce blood vessels and nervous system and inability to undergo complete regeneration (Stampoultzis et al., 2021). Emerging data have indicated that cell-based techniques are one of the most widely used treatments to repair articular cartilage; the complex consists of a cell and biomaterial scaffold that provides the necessary conditions for cell attachment, proliferation, and differentiation and has a better short-term cartilage repair effect than ordinary scaffolds. Currently, collagen and alginate are widely used in cartilage tissue engineering; however, there are no clear reports on the comparison of the two as materials for CSPCs. Therefore, our study focused on comparing collagen and alginate as substrates for chondrogenesis derived from CSPCs cultured *in vivo* to determine whether they are promising biomaterials for cartilage regeneration. To assess the effect on cartilage formation of the two biomaterials, the CSPCs/biomaterial were subjected to chondrocyte differentiation by culturing in the chondrogenic differentiation-inducing solution for 3 weeks followed by transplantation into athymic mice. After 4 weeks, histological analysis was performed to evaluate the capacity of cartilage production. The results showed that type I rat tail collagen had beneficial effects on chondrogenic differentiation of CSPCs.

Collagen is the most abundant protein in mammalian tissues (e.g., cartilage, skin, bone, tendons, blood vessels), with excellent biocompatibility, low antigenicity, low cost, and is available in large quantities (Kisling et al., 2019; Lee et al., 2021). There are more than 12 types of collagen in mammalian tissues (type I, II, III, V, X). Cai et al. (2020) explored the chondrogenic differentiation of BMSCs in type I collagen hydrogel and found that MSCs successfully underwent chondrogenic differentiation. In our study, we selected type I collagen as a biomaterial to compare with alginate. Following gross observation of CSPCs/scaffolds, we observed that type I rat tail collagen and the alginate group could form transparent cartilage-like tissue under the skin of nude mice after 4 weeks. This observation indicated the mechanical performance of type I rat tail collagen and alginate as biomaterials for cartilage regeneration and reflected their biodegradability. Furthermore, we performed histological evaluation to assess the chondroid matrix formation of CSPCs with the help of the two biomaterials. Histological analysis showed that bioactive molecules-pretreated in the type I rat tail collagen/CSPCs group and alginate/CSPCs group formed a relatively mature cartilage structure in which the basic characteristics of cartilage tissue, including cartilage lacuna and cartilage capsule, were clearly visible. Histological and immunofluorescence evaluation of cartilage-specific matrix glycosaminoglycan (GAG) and Col2 further confirmed that type I rat tail collagen had a more beneficial effect than alginate on the chondrogenic differentiation of CSPCs, and the chondrocyte-like cells generated from CSPCs showed similar features to those of mature chondrocytes in terms of cartilage-specific GAG production and Col2 accumulation. Similarly, Miao and colleagues found higher GAG production from chondrogenically stimulated MSCs embedded in collagen scaffolds compared with alginate, which explored four kinds biomaterials for chondrocytes in vitrol (Miao et al., 2018). Also, Jin, et al. found type Ⅰ collagen hydrogel promoted chondrocytes to accumulate more GAG than alginate (Jin and Kim, 2018). Nevertheless, a positive reaction was observed in the non-induced type I rat tail collagen group, indicating a strong support of chondrogenic development by the type I rat tail collagen hydrogels. Regarding the staining reaction, the following mechanisms could explain the results: first, the CSPCs could have spontaneously differentiated into chondrocytes, and second, we obtained the CSPCs through the differential adhesion method, which might have resulted in the CSPCs being mixed with chondrocytes. Accordingly, the cells in type I rat tail collagen exhibited increased gene expression of COL2 and ACAN, suggesting a strong increase in chondrogenic differentiation embedded in the type I rat tail collagen scaffolds. Moreover, gene expression of chondrogenic marker SOX9 was more upregulated in type I rat tail collagen hybrid scaffolds than in alginate. As a key factor in chondrogenic commitment, SOX9 not only maintains chondrocyte morphology, but also accelerates the output of collagen type II and aggrecan (Miao et al., 2018; van Gastel et al., 2020).

Gene expression of COL10 and RUNX2 were upregulated in the alginate biomaterials group compared with the type I rat tail collagen scaffolds group. Type X collagen and runx2 are the index of cartilage tissue hypertrophy and play an important role in endochondral ossification. Their upregulated expression in cartilage tissue suggested cartilage tissue hyperplasia and hypertrophy, which indirectly reflected the biological functional state of chondrocytes. Immunofluorescence analysis of collagen X revealed that it was inhibited in type I rat tail collagen hybrids in coordination with gene expression and the RUNX2 expression level, suggesting that the chondrogenesis of CSPCs maintained a relatively stable phenotype. Moreover, upregulation of gene expression of all chondrogenic differentiation markers and downregulation of chondrocyte hypertrophy in type I rat tail collagen demonstrated that type I rat tail collagen had an advantage over alginate in chondrogenic development from CSPCs. Even though gene expression of COL1 was upregulated in alginate group, we didn’t found positive reaction of collagen Ⅰ in every group, which displayed a negative marker for fibration. To a certain extent, CSPCs displayed a superior chondrogenic capacity.

However, necrosis was observed in all type I rat tail collagen and alginate hybrids, indicated by massive infiltration of inflammatory cells. Several mechanisms could explain this observation: first, the biomaterials degraded too rapidly to collapse, which caused the cells to lose their growth space and resulted in ischemia and hypoxia of the internal cells, and second, a large amount of extracellular matrix accumulated in the superficial coat, hindering nutrient penetration into the inner coat.

The combined scaffolds, either type I rat tail collagen or pure alginate, supported chondrogenic differentiation of CSPCs. Compared with alginate scaffolds, CSPCs embedded in type I rat tail collagen hybrids exhibited more type II collagen and aggrecan accumulation and upregulated gene expression of chondrogenic markers while inhibiting formation of type X collagen and downregulating gene expression of hypertrophic markers. These results demonstrated that type I rat tail collagen could promote chondrogenesis derived from CSPCs, but its degradation rate must be tailored to match the cartilage differentiation rate.

## Data Availability

For the privacy of individuals that participated in the study, the data underlying this article cannot be shared publicly. The data underlying this article will be shared on reasonable request to the corresponding author.
