# Corrigendum: Comparative study of alginate and type I collagen as biomaterials for cartilage stem/progenitor cells to construct tissue-engineered cartilage *in vivo*

**DOI:** 10.3389/fbioe.2024.1417779

**Published:** 2024-06-13

**Authors:** Xiaodie Zhang, Lin Qi, XiaoGang Chen, Yongxian Lai, Kai Liu, Ke Xue

**Affiliations:** ^1^ Department of Dermatologic Surgery, Shanghai Skin Disease Hospital, Tongji University School of Medicine, Shanghai, China; ^2^ Department of Radiology, Huadong Hospital Affiliated to Fudan University, Shanghai, China; ^3^ Department of Plastic and Reconstructive Surgery, Shanghai 9th People’s Hospital, Shanghai Jiao Tong University School of Medicine, Shanghai, China; ^4^ Department of Burn and Plastic Surgery, Hainan Western Central Hospital, Shanghai, China

**Keywords:** alginate, type Ⅰ collagen, cartilage, cartilage stem/progenitor cells, tissue engineering

In the published article, there was an error in the **Author list**, and author Lin Qi was erroneously excluded as the **co-first author**. The corrected author list appears below.

“Xiaodie Zhang^1,†^, Lin Qi^2,†^, XiaoGang Chen and Yongxian Lai^1,^*, Kai Liu^3,^*, and Ke Xue^3,4,^*”

“†These authors share first authorship”

The authors apologize for this error and state that this does not change the scientific conclusions of the article in any way. The original article has been updated.

